# A Quantitative Measure of Pain with Current Perception Threshold, Pain Equivalent Current, and Quantified Pain Degree: A Retrospective Study

**DOI:** 10.3390/jcm12175476

**Published:** 2023-08-23

**Authors:** So Yeon Lee, Joong Baek Kim, Jung Woong Lee, A Mi Woo, Chang Jae Kim, Mee Young Chung, Ho Sik Moon

**Affiliations:** Department of Anesthesiology and Pain Medicine, Eunpyeong St. Mary’s Hospital, College of Medicine, The Catholic University of Korea, Seoul 03312, Republic of Korea; soyeon0719@naver.com (S.Y.L.); paparainbow@naver.com (J.B.K.); jwgoethe86@gmail.com (J.W.L.); 01036197939@naver.com (A.M.W.); ksw070591@catholic.ac.kr (C.J.K.); jhjs0806@catholic.ac.kr (M.Y.C.)

**Keywords:** pain, pain measurement, numeric rating scale, pain threshold, parameter, pain deception, visual analog scale

## Abstract

**Background:** As a subjective sensation, pain is difficult to evaluate objectively. The assessment of pain degree is largely dependent on subjective methods such as the numeric rating scale (NRS). The PainVision^TM^ system has recently been introduced as an objective pain degree measurement tool. The purpose of this study was to analyze correlations between the NRS and the current perception threshold (CPT), pain equivalent current (PEC), and quantified pain degree (QPD). **Methods:** Medical records of 398 subjects who visited the pain clinic in a university hospital from March 2017 to February 2019 were retrospectively reviewed. To evaluate the pain degree, NRS, CPT, PEC, and QPD were measured. Subjects were categorized into two groups: the Pain group (*n* = 355) and the No-pain group (*n* = 43). **Results:** The NRS showed a negative correlation with CPT (R = −0.10, *p* = 0.054) and a positive correlation with QPD (R = 0.13, *p* = 0.008). Among various diseases, only spinal disease patients showed a negative correlation between CPT and NRS (R = −0.22, *p* = 0.003). Additionally, there were significant differences in CPT and QPD between the Pain and No-pain groups (*p* = 0.005 and *p* = 0.002, respectively). **Conclusions:** CPT and QPD measured using the PainVision^TM^ system could be used to estimate pain intensity and the presence of pain. These parameters would be considered useful for predicting pain itself and its intensity.

## 1. Introduction

The International Association for the Study of Pain (IASP) defines pain as “An unpleasant sensory and emotional experience associated with, or resembling that associated with, actual or potential tissue damage,” and it is expanded upon with the addition of six key notes and the etymology of the word pain for further valuable context [1]. Since pain affects patients’ daily living and quality of life, it is one of major healthcare problems worldwide. It is also a socioeconomic burden [2,3]. Currently, the Korean society is afflicted with rapid aging. Proper management and monitoring of these demographic changes are critical [4]. Pain is often underdiagnosed and undertreated, especially in older patients, due to difficulties in evaluating the intensity of pain and the effectiveness of treatment [5]. 

An objective assessment of pain remains a challenge for doctors because of its nature of “subjectivity”, which can be easily influenced by various factors, including individual experiences, cultural backgrounds, and psychological conditions. For example, many people endure pain in Korea because the culture teaches that patience is a virtue [6]. Alongside physical predictors, there is increasing recognition of psychosocial factors in chronic pain. Affective factors such as depression, anxiety disorders, stress, negative thoughts, neuroticism, and catastrophizing can predict future pain. Broader social factors such as low perceived social acceptance and poor social relations have also been identified as predictors [7,8,9]. On the contrary, engaging in vigorous weekly activity and cultural engagement was protective against the development of chronic pain [10]. 

Several self-reporting measurement tools such as the visual analog scale (VAS) and the numeric rating scale (NRS) are often used for assessing pain intensity. Patients score themselves using either scale in the range of 0 to 10 corresponding to ‘no pain’ and ‘the worst pain imaginable’, respectively. The VAS and NRS are among the popular quantitative scales. They match quite well with acute pain degrees. Both are also known to be superior to the four-point verbal categorical rating scale (VRS) (none, mild, moderate, and severe) [11]. For evaluating pain quality, many questionnaires are also used. Such values have the advantage of being easy to understand but research results on their validity and reliability are not always consistent. Sebastian et al. reported that the PainDETECT questionnaire (PD-Q) and NRS scores significantly correlated at the baseline visit and the 1-month follow-up visit in chronic pain patients; therefore, it is a useful screening tool for clinicians to use when diagnosing or predicting the treatment response of neuropathic pain [12]. On the other hand, according to a systematic review of the criterion validity and reliability of neuropathic pain screening questionnaires, many of them had limited measurement properties. While the Douleure Neuropathique 4 (DN4) and Neuropathic Pain Questionnaires had the greatest number of satisfactory measurement properties and were most suitable for clinical use, others including ID Pain, LANSS, etc., were unsatisfactory [13].

As pain is always subjective, a patient’s report of pain should be accepted at face value in the absence of evidence to the contrary. In order to compensate for these shortcomings, efforts are being made to develop objective pain diagnostic tools, biomarkers, and imaging [14]. Quantitative sensory testing (QST) is a panel of diagnostic tools that evaluates somatosensory functions, including hot, cold, vibration, touch, pinprick, and pressure pain, and investigates the severity of clinical signs using calibrated stimuli and subjective perception thresholds [15,16]. It has the advantage of providing objective and quantitative outcomes of the sensory dysfunction including chronic pain. On the other hand, one of the disadvantages is that it takes about an hour to complete the QST profile. During the test, mechanical pain sensitivity increases significantly, which can cause hyperalgesia in healthy volunteers [17]. Another drawback is that the reliability and validity of the test are low. The overall consistency of the results from the first and the second tests is low [18].

**Figure 1:**
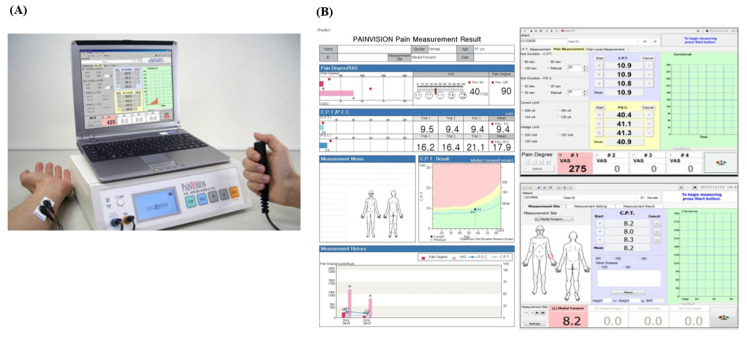
PainVision^TM^ system and a result paper. (**A**) The PainVision^TM^ system and (**B**) a result paper from the PainVision^TM^ system.

PainVision^TM^ (PS-2100, supplier: Nipro, maker: Osachi Corporation, Osaka, Japan) (Figure 1) is an equipment designed to quantitatively assess a patient’s pain by converting it into a heterogeneous sensation using electric stimuli. The pain degree is calculated and notated as two parameters: the current perception threshold (CPT) and the pain equivalent current (PEC). The CPT is defined by the lowest electrical current at which the patient detects the stimuli [19]. The PEC is defined by the lowest electrical current at which the patient detects the pain. The quantified pain degree (QPD) is calculated with the following equation: QPD = (PEC − CPT)/CPT × 100. By digitizing pain assessment (especially evaluation of central sensitizing pain) and treatment progress, it is easier to adjust dosage of analgesic drugs and share a patient’s “pain” with other physicians. Further, it also can be used for the early diagnosis of neuropathic pain ahead of subjective symptoms. This non-invasive and quick test proved to be an objective screening method for diabetic peripheral neuropathy in busy clinics, ensuring adherence to the current unfulfilled recommendations for annual assessments for all diabetic patients [20].

In this study, we hypothesized that the CPT, PEC, and QPD would be correlated with pain intensity. Thus, the primary purpose of this study was to evaluate correlations between the NRS and CPT, PEC, and QPD. The secondary purpose was to compare the relationship between the NRS, CPT, PEC, and QPD in each disease group and compare values of the CPT, PEC, and QPD between the Pain and No-pain groups.

## 2. Materials and Methods

### 2.1. Study Design and Setting

This was a retrospective observational study that used medical records. It was approved by the Institutional Review Board of Yeouido St. Mary’s Hospital Ethics Committee (Approval number: SC19RESI0078) on 9 July 2019. Medical records of patients who visited the pain clinic in a university hospital from March 2017 to February 2019 were retrospectively reviewed.

### 2.2. Data Measurements and Analysis

The PainVision^TM^ system was used, which is composed of an electrical pulse generator, a monitor, a computer, two disposable skin electrodes, and a handle with a button (Figure 1A). Subject took a sitting position with their forearms supinated on a table. Researchers attached skin electrodes to the subject’s forearm (ulnar side) at 1 cm from the midline, which transmitted an electrical current (Figure 1A). The parameters were set as the default: the measuring time was 100 s (s), the limiting current was 256 microamperes (μA), and the limiting current was 100 volts (V). We turned on the PainVision^TM^ system and entered the patient demographic information to start the measurement [21]. Subjects were asked to push the button on the handle when they first felt electrical stimulation or electrical pain while gradually increasing the current pulses (50 Hz of frequency, 0–150 μA of pulse amplitude, and 0.3 ms of pulse width) through the skin electrodes. The former was defined as CPT, while the latter was defined as PEC. Each parameter was measured three times, and their mean value was calculated (Figure 1B). The QPD value was defined with the following formula: QPD = (PEC − CPT)/CPT × 100. The physiopathological foundations of the signal recording of the PainVision^TM^ system are not fully understood because pain is a complex sensory experience that is mediated by the central nervous system and peripheral nervous system. The PainVision^TM^ system can only detect the electrical current, which is strong enough to activate Aβ and Aδ fibers, depending on the electrical stimuli on the skin [22].

### 2.3. Statistical Methods

All data are expressed as mean ± standard deviation or the number of samples. NRS, CPT, PEC, and QPD were compared separately between the Pain and No-pain groups using an unpaired *t*-test. The CPT and QPD values for the Pain group and the No-pain group were expressed as the median and IQR using a box plot. Correlations between the QPD and NRS were analyzed using a Pearson correlation analysis. In addition, a multiple regression analysis was used to predict pain deception using a QPD value. Statistical analyses were performed using the IBM SPSS Statistics for Windows program, version 22.0 (IBM Corp., Armonk, NY, USA). *p* < 0.05 was considered statistically significant. 

## 3. Results

A total of 398 patients who had been examined for CPT, QPD, and PEC with the PainVision^TM^ system were included in this study. In these enrolled subjects, spinal disease (45.2%) was the most common, followed by neuropathic pain disease (18.3%), CRPS (9.3%), and so on (Table 1). 

### 3.1. Correlations between the NRS and Parameters of the PainVision^TM^ System

**Figure 2:**
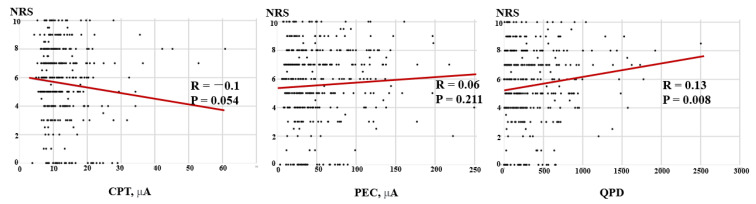
Correlations between the NRS and CPT, QPD, and PEC for all subjects. The scatter diagram shows a negative correlation between the NRS and CPT but a positive correlation between the NRS and QPD for all patients. NRS: numeric rating scale; CPT: current perception threshold; PEC: pain equivalent current; QPD: quantified pain degree.

**Figure 3:**
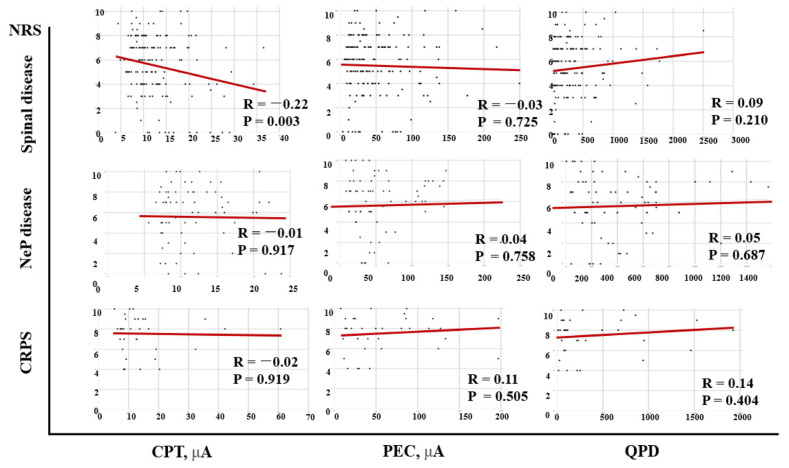
Correlations between the NRS and CPT, QPD, and PEC according to diseases. There is a negative correlation between the NRS and CPT in spinal diseases subjects. NRS: numeric rating scale; CPT: current perception threshold; PEC: pain equivalent current; QPD: quantified pain degree; CRPS: complex regional pain syndrome; NeP: neuropathic pain.

Correlations between the NRS and CPT and QPD of the patients are shown in Figure 2. The NRS showed a negative correlation with CPT (R = −0.10, *p* = 0.054) but a significant positive correlation with QPD (R = 0.13, *p* = 0.008). In spinal disease patients (45.2%), there was a negative correlation between NRS and CPT (R = −0.22, *p* = 0.003) (Figure 3). 

### 3.2. Differences between the Pain and No-Pain Groups

**Figure 4:**
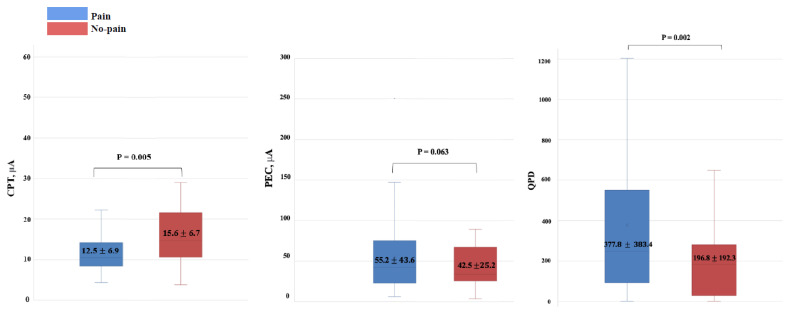
Box plots for the CPT and QPD values comparing the Pain and No-pain groups. There are statistically significant differences in the CPT and QPD values between the Pain and No-pain groups. CPT: current perception threshold; QPD: quantified pain degree.

The secondary analysis was conducted using the Pain and No-pain groups depending on the presence or absence of pain (Table 2). There were significant differences in the CPT and QPD between the Pain and No-pain groups (Figure 4).

## 4. Discussion

It would be worth acknowledging previous work that has demonstrated that the QST, pain pressure threshold (PPT), and other measures do not necessarily align with numerical ratings of pain intensity [23,24]. In this retrospective study, only the QPD showed a significant correlation with the NRS, whereas the CPT and PEC did not. There were also significant differences in the CPT and QPD between the Pain and No-pain groups. These results show that quantitative pain parameters using the electrical stimulus of the PainVision^TM^ system such as the QPD and CPT can play a supportive role in estimating pain intensity and pain presence. Objective assessments would be particularly valuable for non-verbal individuals or people with cognitive decline.

### 4.1. Reliability of Parameters Using Electrical Stimulation

There are a few studies about the reliability of parameters using electrical stimulation of the PainVision^TM^ system or the Neurometer^TM^ system as diagnostic markers for chronic pain [25,26,27,28,29]. In healthy volunteers, the reproducibility of the CPT with the Neurometer^TM^ system at 250 or 2000 Hz was acceptable, with intra-class correlation coefficients (ICCs) of 0.615 and 0.735 at 250 and 2000 Hz, respectively. However, the reproducibility of the CPT with the Neurometer^TM^ system at 5 Hz was not acceptable (ICCs: 0.292 to 0.318) [28]. To assess intra-oral sensitivity to electrical stimulation in the mental foramen region, ICCs for CPT and pain threshold (PT) were >0.8, showing appropriate reliability [25]. Similarly, CPTs and PTs obtained from the oral cavity, hand, and foot showed reliable results (Cronbach’s α coefficients > 0.78 at 5 Hz, 250 Hz, and 2000 Hz) [30]. In lower back pain patients (*n* = 25) using the PainVision^TM^ system, test–retest reliability was significant (ICC = 0.967, *p* < 0.001) [31].

### 4.2. Validity of Electrical Parameters as Quantitative Diagnostic Markers for Pain

The results of this study revealed that the NRS was correlated with the QPD and that there were differences in the CPT and QPD between the Pain and No-pain groups. However, this does not mean that the CPT or QPD can quantify pain intensity. In the scatter diagram showing the relationship between the NRS and CPT/PEC/QPD (Figure 2), there were a few outliers for the parameter that could affect the correlation coefficient. Various analyses were performed to determine whether outliers had a significant effect on the results. It was confirmed that the robustness of the results of this paper was guaranteed and that the influence of outliers was not significant. Even after removing those outliers, the statistical significance of the analysis results was maintained. Since the sample size was not large enough, data that appeared to be outliers could have a large impact on the model, which could change the statistical validation results. However, since the pattern for the entire data showed a similar tendency to statistical results, it was not necessary to define data that looked like outliers as outliers. We tested the robustness of the model results in this way and concluded that the current analysis results were not problematic. To date, papers evaluating the validity of CPT/PEC/QPD as a quantitative pain assessment tool are insufficient and have inconsistent results. Inoue et al. reported that there was no correlation between the CPT measured with the PainVision^TM^ system and the VAS score in cervical myelopathy patients (*n* = 158) or healthy volunteers (*n* = 100). However, the CPT showed significant associations with other variables, including the Japanese Orthopaedic Association (JOA) score, the JOA cervical myelopathy evaluation questionnaire (JOAMEQ), and the quality of life [32]. Kim et al. reported that although there was a significant correlation between the QPD and MPQ, there was no correlation between the QPD and VAS (Spearman rank correlation coefficients = 0.240, *p* = 0.248) [31]. On the other hand, Wang et al. reported that the QPD and VAS in postherpetic neuralgia patients (*n* = 40) were significantly correlated with both persistent pain (r = 0.453, *p* = 0.008) and breakthrough pain (r = 0.64, *p* = 0.001) [33]. Ohtori et al. reported that the QPD had a positive correlation with the NRS (r = 0.40, *p* = 0.03) in 89 lower back pain patients [22]. Similarly, in colorectal cancer patients (*n* = 64) who received chemotherapy, the partial correlation coefficient between the VAS and QPD after adjusting for sex and subject was 0.274 (*p* = 0.0003) [34].

### 4.3. CPT and the Type of Pain Disease

Although the sample size for each pain disease was not sufficient, correlations between the NRS and PainVision^TM^ system’s parameters such as the CPT, PEC, and QPD were different according to the type of pain disease. A statistically significant negative correlation between the NRS and CPT was only found in the spinal disease group. No study has reported differences in the CPT depending on the type of pain disease. Especially, since research using the PainVision^TM^ system has not yet been applied to various diseases, the difference between diseases is currently unknown. Usually, the CPT value obtained using the Neurometer^TM^ system is measured at three frequencies: 2000 Hz for Aβ, 250 Hz for Aδ, and 5 Hz for C fiber [35], which is different from the method of the PainVision^TM^ system, i.e., 50 Hz of frequency. Based on this fact, the bias of this study’s results can be interpreted as follows. First, even for the same disease, test results can be different depending on which sensory nerves are more preferentially involved. Because this is a retrospective study, the authors could not control subjects according to their symptoms usually associated with the type of sensory nerves. Second, since the PainVision^TM^ system is measured in the range of 50 Hz, it is difficult to compare its results with the results from studies using the Neurometer^TM^ system.

### 4.4. Confounding Factors That Can Affect the CPT and PEC

Acquisition of the CPT and PEC using the PainVision^TM^ system is similar to the CPT measured with the Neurometer^TM^ in terms of using an electrical stimulus [35]. Several studies have reported confounding factors that can affect the CPT using the Neurometer^TM^. Generally, the CPT is known to be higher in older individuals and males. Sex differences in the CPT are especially associated with body fat and body water percentages, which can affect the sensitivity for perceiving the electrical stimulation [36,37]. Accompanying diseases including diabetes and chronic alcoholism, which can easily lead to polyneuropathy and psychiatric diseases such as chronic schizophrenia, can influence the result, which elevates the CPT [38,39]. 

### 4.5. Limitation of This Study

This study has several limitations. First, confounding factors, including gender, age, psychological stress, concomitant diseases, taking drugs, and so on, that could affect the response of subjects to electrical stimuli were not considered thoroughly in this study. Second, we could not thoroughly compare differences in the CPT, PEC, and QPD according to disease groups because the sample size was uneven and small for each disease group. There were less than 10 participants in the OA, failed back surgery syndrome, fibromyalgia, headache, and cancer groups. Lastly, the sample size for the No-pain group (namely, healthy subjects) was not sufficient, and the NRS distribution in the Pain group was not even. Hence, we could not estimate the cut-off value for the diagnosis of pain itself or for dividing the NRS using parameters such as the CPT, PEC, and QPD. 

### 4.6. Prospects of Pain Assessment

In conclusion, electrical parameters, especially the CPT and QPD, measured using the PainVision^TM^ system can be used to estimate pain intensity and the presence of pain. These parameters are very useful to predict pain itself and its intensity. The objective study of pain is now also focusing on other elements such as the autonomic nervous system (ANS) or physiological signals, as well as behavioral aspects. Changes in the ANS have important prognostic and diagnostic value and can be used to assess stress levels or pain. For example, electrodermal activity (EDA) and heart rate variability (HRV) are useful parameters [40,41]. Multimodal approaches combining the PainVision^TM^ system with various biofeedback parameters (e.g., EDA, HRV, brain activity, muscle activity, respiration, skin temperature) and deep learning-based artificial intelligence will be of great help in clinical fields by automating and digitizing objective pain assessments [42,43]. They will also help develop wearable sensors and devices [44]. Further research aimed at the development of objective, standardized, and generalizable instruments for pain assessment in various clinical contexts is needed.

## Figures and Tables

**Table 1:** Demographic data of the subjects included in this study.

		Subjects
Total numbers		398 (100)
Age, years		54.4 ± 16.0
Gender, n (%)	Male	158 (39.7)
	Female	240 (60.3)
Diagnosis, n (%)	Spinal disease	180 (45.2)
	NeP disease	73 (18.3)
	CRPS	37 (9.3)
	Normal	26 (6.5)
	OA	10 (2.5)
	FBSS	9 (2.3)
	Fibromyalgia	7 (1.8)
	Headache	7 (1.8)
	Cancer	2 (0.5)
	Other	47 (11.8)
Duration of the disease, n (%)	<3 months	323 (81.1)
	≥3 months	75 (18.9)

NeP: neuropathic pain; CRPS: complex regional pain syndrome; FBSS: failed back surgery syndrome; OA: osteoarthritis. All values are presented as the sample number and percentage, n (%), or mean ± standard deviation.

**Table 2:** The NRS, CPT, QPD, and PEC data for the Pain and No-pain groups.

	Pain Group	No-Pain Group	*p*-Value
Subjects, n (%)	355 (89.2)	43 (10.8)	
NRS	5.5 ± 2.8	0	
CPT, µA	12.5 ± 6.9	15.6 ± 6.7	0.005
QPD	377.8 ± 383.4	196.8 ± 192.3	0.002
PEC, µA	55.2 ± 43.6	42.5 ± 25.2	0.063

NRS: numeric rating scale; CPT: current perception threshold; PEC: pain equivalent current; QPD: quantified pain degree. All values are presented as the sample number and percentage, n (%), or mean ± standard deviation.

## Data Availability

The datasets generated and/or analyzed during the current study are not publicly available because disclosing patients’ personal information is against the law, but de-identified datasets are available from the corresponding author on reasonable request.
